# Podosomes in migrating microglia: components and matrix degradation

**DOI:** 10.1186/1742-2094-9-190

**Published:** 2012-08-08

**Authors:** Catherine Vincent, Tamjeed A Siddiqui, Lyanne C Schlichter

**Affiliations:** 1 Toronto Western Research Institute, Room MC9-417, University of Toronto, 399 Bathurst Street, Toronto, ON, M5T 2 S8, Canada; 2 Department of Physiology, University of Toronto, Toronto, Canada

**Keywords:** Neuroinflammation, Microglia migration, Cell adhesion, Cell invasion, Extracellular matrix degradation, Signaling complex, Lamellipodia, Invadopodia, Invadosomes, Podosomes

## Abstract

**Background:**

To perform their functions during development and after central nervous system injury, the brain’s immune cells (microglia) must migrate through dense neuropil and extracellular matrix (ECM), but it is not known how they degrade the ECM. In several cancer cell lines and peripheral cells, small multi-molecular complexes (invadopodia in cancer cells, podosomes in nontumor cells) can both adhere to and dissolve the ECM. Podosomes are tiny multi-molecular structures (0.4 to 1 μm) with a core, rich in F-actin and its regulatory molecules, surrounded by a ring containing adhesion and structural proteins.

**Methods:**

Using rat microglia, we performed several functional assays: live cell imaging for chemokinesis, degradation of the ECM component, fibronectin, and chemotactic invasion through Matrigel™, a basement membrane type of ECM. Fluorescent markers were used with high-resolution microscopy to identify podosomes and their components.

**Results:**

The fan-shaped lamella at the leading edge of migrating microglia contained a large F-actin-rich superstructure composed of many tiny (<1 μm) punctae that were adjacent to the substrate, as expected for cell–matrix contact points. This superstructure (which we call a podonut) was restricted to cells with lamellae, and conversely almost every lamella contained a podonut. Each podonut comprised hundreds of podosomes, which could also be seen individually adjacent to the podonut. Microglial podosomes contained hallmark components of these structures previously seen in several cell types: the plaque protein talin in the ring, and F-actin and actin-related protein (Arp) 2 in the core. In microglia, podosomes were also enriched in phosphotyrosine residues and three tyrosine-kinase-regulated proteins: tyrosine kinase substrate with five Src homology 3 domains (Tks5), phosphorylated caveolin-1, and Nox1 (nicotinamide adenine dinucleotide phosphate oxidase 1). When microglia expressed podonuts, they were able to degrade the ECM components, fibronectin, and Matrigel™.

**Conclusion:**

The discovery of functional podosomes in microglia has broad implications, because migration of these innate immune cells is crucial in the developing brain, after damage, and in disease states involving inflammation and matrix remodeling. Based on the roles of invadosomes in peripheral tissues, we propose that microglia use these complex structures to adhere to and degrade the ECM for efficient migration.

## Background

Regulated invasion of cells through tissue is crucial for many physiological processes, including inflammation and wound healing, but these processes also drive tumor cell invasion and metastasis [1]; there is thus considerable impetus to understand the underlying mechanisms. As the innate immune cells of the brain, microglia perform many roles – from debris clearance to secretion of growth factors and potentially toxic molecules. Their capacity to migrate is crucial to both central nervous system (CNS) development and responses to damage in the adult brain. For instance, early in development, precursor cells migrate into the brain parenchyma where they differentiate into mature microglia [2-4], and microglia are thought to be replenished throughout life from invasion of monocyte-lineage cells [5,6]. In the perinatal period, microglia are amoeboid and highly migratory [6,7]. In the healthy adult, microglia are ramified and relatively quiescent but their cell processes continually extend and retract without overall cell displacement [4,6,7]. One of the earliest responses to acute CNS injury is microglia translocation to the site of damage [8] and rapid process extension [6,7]. While conducting *in vivo* studies, we were struck by the relatively long distances over which microglia migrate after optic nerve transection [9], intracerebral hemorrhage [10] and transient ischemic stroke [11]. Although microglia migration has been studied *in vitro*, in the CNS they must traverse the densely packed neuropil and extracellular matrix (ECM) that occupy a large proportion of the brain volume [12], and the underlying mechanisms are not known.

Cell migration includes undirected motility (chemokinesis), movement toward a soluble chemoattractant molecule (chemotaxis) and migration along a gradient of nonsoluble substrate (haptotaxis). Migrating cells are polarized along the axis of movement, and often display a fan-shaped lamella with thin (0.4 to 1.0 μm) protrusions (lamellipodia) at the leading edge, and a uropod at the rear [13]. As a cell advances, newly extended protrusions adhere to the extracellular substrate using integrins, while traction force is generated by interaction of myosin II with actin filaments that are attached through adaptor proteins to the integrins [14]. The adhesion sites are reassembled in newly protruded regions and disassembled at the uropod; mechanisms for their rapid turnover are therefore needed. In addition, migrating cells require mechanisms for degrading and remodeling the ECM, whether they are moving within a tissue or invading across blood vessels, similar to extravasating immune cells and metastatic cancer cells.

Some cancer cells were recently found to form invadopodia, which are small (about 8 × 5 μm) F-actin-rich, perinuclear structures that are enriched in matrix metalloproteases, can dissolve the ECM, and are thought to facilitate cell invasion [15,16]. A related structure in nontumor cells, the podosome, was described as 0.4 to 1 μm diameter, electron-dense and vinculin-rich membrane protrusions in Src-transformed fibroblasts [17,18], and in the sealing zone of osteoclasts where these structures mediate bone resorption [19]. Podosomes are abundant (100 or more per cell), highly dynamic (turnover times of 2 to 12 minutes), and known to degrade the substrates gelatin and fibronectin [16,20,21]. Together, podosomes and invadopodia are subcellular structures with the unique ability to adhere to and degrade the ECM. The present study arose from our observation that migrating microglia have a large donut-shaped structure in the lamella at the leading edge, composed of hundreds of tiny F-actin-rich punctae. We show that these punctae are individual podosomes, which express several hallmark molecules in the classical core and ring arrangement, and that microglia bearing podosomes can degrade the ECM.

## Methods

### Cells

All procedures on animals were approved by the University Health Network Animal Care Committee, in accordance with guidelines from the Canadian Council on Animal Care. Microglia cultures were prepared from 1-day-old to 2-day-old Sprague–Dawley rat pups (Charles River, St-Constant, PQ, Canada) using our standard protocols, which yield ≥99% purity [22,23]. In brief, the brain was dissected, minced in cold minimal essential medium (MEM; Invitrogen, Carlsbad, CA, USA), centrifuged (300 × *g*, 10 minutes), and resuspended in MEM with 10% fetal bovine serum (FBS; Wisent, St-Bruno, PQ, Canada) and 0.05 mg/ml gentamycin (Invitrogen). The dissociated cells were then seeded in 75 cm^2^ flasks and incubated at 37°C and 5% CO_2_. After 48 hours the medium was changed to remove cellular debris and nonadherent cells, and after a further 4 to 5 days in mixed culture the microglia were harvested by shaking the flasks for 2 to 4 hours on an orbital shaker at 65 rpm (37°C, 5% CO_2_). After centrifuging the microglia-rich supernatant (300 × *g*, 10 minutes), the cell pellet was resuspended in fresh MEM (with 2% FBS). Microglia were seeded onto UV-irradiated 15 mm glass coverslips (Fisher Scientific, Ottawa, ON, Canada) at 50,000 or 60,000 cells per coverslip in 12-well plates, and cultured for 1 to 2 days in MEM (2% FBS). Importantly, we find under these growth conditions that the microglia are relatively resting [24]. In one experiment, microglia were plated onto Ultra-Web™ (VWR, Mississauga, ON, Canada), which is a three-dimensional (3D) mesh of electrospun polyamide nanofibers.

### Live cell imaging

Microglia were plated at 60,000 cells per 35 mm glass-bottom culture dish (MatTek Corporation, Ashland, MA, USA), and were cultured for 2 days (MEM, 2% FBS). Live microglia were imaged for up to 1 hour using a Zeiss Axiovert 200 M microscope (Zeiss, Toronto, ON, Canada), an ORCA-ER camera (Hamamatsu Corporation, Bridgewater, NJ, USA), Axiovision software (Zeiss) and a Neue LiveCell™ stage top incubator (Pathology Devices, Westminster, MD, USA) to maintain 37°C and 5% CO_2_.

### Immunocytochemical analysis

Microglia were seeded at 50,000 to 60,000 cells per 15 mm glass coverslip, and were cultured for 2 to 3 days in MEM with 2% FBS. The standard fixative (except for nicotinamide adenine dinucleotide phosphate oxidase 1 (Nox1), see below) was 4% paraformaldehyde (Electron Microscopy Sciences, Hatfield, PA, USA) at room temperature for 15 minutes. Cells were then permeabilized with 0.2% Triton X-100 for 5 minutes and washed in PBS (three times, 5 minutes each), and then nonspecific antigens were blocked with 4% donkey serum for 1 hour. Because Nox1 antibody stained poorly after paraformaldehyde fixation, microglia were fixed and permeabilized with HPLC-grade methanol (5 minutes, –20°C) that had been prechilled overnight to −40°C. After methanol fixation, F-actin could not be labeled with phalloidin [25].

All antibodies (Table 1) were diluted in 2.5% donkey serum and centrifuged before use (8,200 × *g*, 10 minutes) to precipitate any aggregated antibody that might be present. Microglia were incubated with one or two primary antibodies overnight at 4°C, washed (three times, 5 minutes each) and blocked with 4% donkey serum for 1 hour, and were then incubated with a donkey secondary antibody for at least 1 hour before washing (three times, 10 minutes each). Negative controls were prepared using the same protocol, but omitting each primary antibody. F-actin was visualized by incubating cells (15 minutes, room temperature in the dark) with Alexa 488-conjugated phalloidin (1:50 in blocking solution; Invitrogen). Cell nuclei were labeled with 4′,6-diamidino-2-phenylindole (1:3,000 in PBS, 5 minutes). After washing (three times, 5 minutes each), cells on coverslips were mounted on glass slides either with 50% glycerol in PBS, which produced the lowest background, or with VectaShield™ (Vector Labs, Burlington, CA, USA) or Dako mounting medium (Dako, Glostrup, Denmark), which yielded more stable signals for longer imaging (Z-stacks).

**Table 1:** Antibodies and stains used

**Antibody/stain (species, type)**	**Concentration**	**Source**
Phalloidin 488 or 350 (labels F-actin)	1:50	Invitrogen (Carlsbad, CA, USA)
DAPI (4′,6-Diamidino-2-phenylindole; labels DNA)	1:3,000	Invitrogen
Talin1/2 (mouse monoclonal)	1:100	Abcam (Cambridge, MA, USA)
Arp2 (actin-related protein 2) (rabbit polyclonal)	1:100	Santa Cruz Biotechnology (Santa Cruz, CA, USA)
Tks5 (tyrosine kinase substrate with five Src homology 3 domains) (rabbit polyclonal)	1:100	Santa Cruz Biotechnology
Phospho-caveolin 1 (p-Tyr^14^Cav1)	1:100	Signalway Antibody
(rabbit polyclonal)		(Pearland, TX, USA)
Anti-phosphotyrosine (mouse monoclonal)	1:100	Abcam
Nox1 (nicotinamide adenine dinucleotide phosphate oxidase 1) (goat polyclonal)	1:100	Santa Cruz Biotechnology
DyLight donkey anti-rabbit 488 or 594 (secondary)	1:250	Jackson Immunoresearch (West Grove, PA, USA)
DyLight donkey anti-mouse 488 or 594 (secondary)	1:250	Jackson Immunoresearch
Donkey anti-goat FITC (secondary)	1:250	Jackson Immunoresearch

Images were acquired with an Axioplan 2 widefield epifluorescence microscope equipped with an Axiocam HRm digital camera, and were analyzed with Axiovision 4.6 software (all Zeiss) or ImageJ (version 1.41o [26]). For many images, we acquired Z-stacks through the entire cell (that is, high-magnification epifluorescence images through the Z-axis at 200 nm increments). These images were then deconvolved using a theoretical point spread function and the constrained iterative maximum-likelihood algorithm in Axiovision 4.7 software (Zeiss). 3D reconstructions were made with Imaris software (Bitplane Scientific Software, Zurich, Switzerland). Deconvolution reduces noise and distortions introduced during image acquisition by using information about the optical system; that is, the type of objective lens, the refractory index of the immersion medium, and the point spread function of light above and below the plane of focus (reviewed in [27]). Cell autofluorescence and nonspecific staining were subtracted using the same imaging and acquisition settings on cells exposed to secondary antibodies alone. When constructing Z-stacks, the automated correction algorithm was used to compensate for fluorescence decay during repeated exposures.

### Chemotaxis, substrate degradation and invasion assays

#### Chemotaxis

Microglia were suspended in MEM with 2% FBS, and 150 μl cell suspension (2 × 10^4^ cells) was added to the upper well of each Transwell™ insert (VWR), which had an uncoated filter bearing pores 8.0 μm in diameter. The lower well contained MEM with 2% FBS, with or without 300 μM ATP. The entire chamber was incubated for 24 hours (37°C, 5% CO_2_). Microglia on the upper side of each filter were removed with a Q-tip, and then the filter was fixed in 4% paraformaldehyde, rinsed with PBS, stained with 0.5% crystal violet for 2 minutes, and again rinsed with PBS. The number of cells that had migrated to the underside was counted (five fields per filter) under phase contrast at 40× magnification using an Olympus CK2 inverted microscope (Olympus, Tokyo, Japan).

#### Substrate degradation

The most common assay for studying ECM degradation by podosomes (and invadopodia) employs fluorescent-labeled fibronectin, either coated directly onto glass coverslips or on a layer of gelatin. ECM degradation is monitored as loss of fluorescence. We labeled bovine fibronectin (Sigma-Aldrich) using the AlexaFluor 488 Protein Labeling Kit (Invitrogen), after which the conjugated protein was separated from unconjugated dye on a column. Purified AlexaFluor 488-conjugated fibronectin was diluted in PBS (~2 μg/ml) and incubated with glass coverslips (150 μl per slip overnight at 37°C). After the solution was aspirated off, microglia were seeded onto the fibronectin-coated coverslips (50,000 cells per coverslip) and incubated for 2.5 to 24 hours. In some experiments, 0.04% Trypan Blue dye (Invitrogen) in PBS was added to quench extracellular fluorescence. Fixation and staining then proceeded as described earlier in Immunocytochemical analysis.

#### Invasion

Microglial invasion through a basement membrane type of ECM (Matrigel™, which is secreted by mouse sarcoma cells) was quantified using 24-well BD BioCoat Matrigel™ Invasion Chambers (BD Biosciences, Mississauga, ON, Canada). The filters, which bore pores 8.0 μm in diameter coated with Matrigel™, were rehydrated for 1 hour at 37°C with 500 μl medium (MEM, 2% FBS). The solution was then replaced with 500 μl fresh MEM (with 2% FBS) containing 2 × 10^4^ microglia. The lower well of each chamber contained 500 μl medium (MEM, 2% FBS), with or without 300 μM ATP. Microglia were incubated (24 hours, 37°C, 5% CO_2_) and counted as above (see Chemotaxis).

### Statistical analysis

Quantitative data are presented as the mean ± standard error of the mean. One-way analysis of variance was followed by a *post-hoc* Tukey’s test, and results are considered significant if *P* < 0.05.

## Results

### Cultured rat microglia display chemokinesis, chemotaxis and invasion

**Figure 1:**
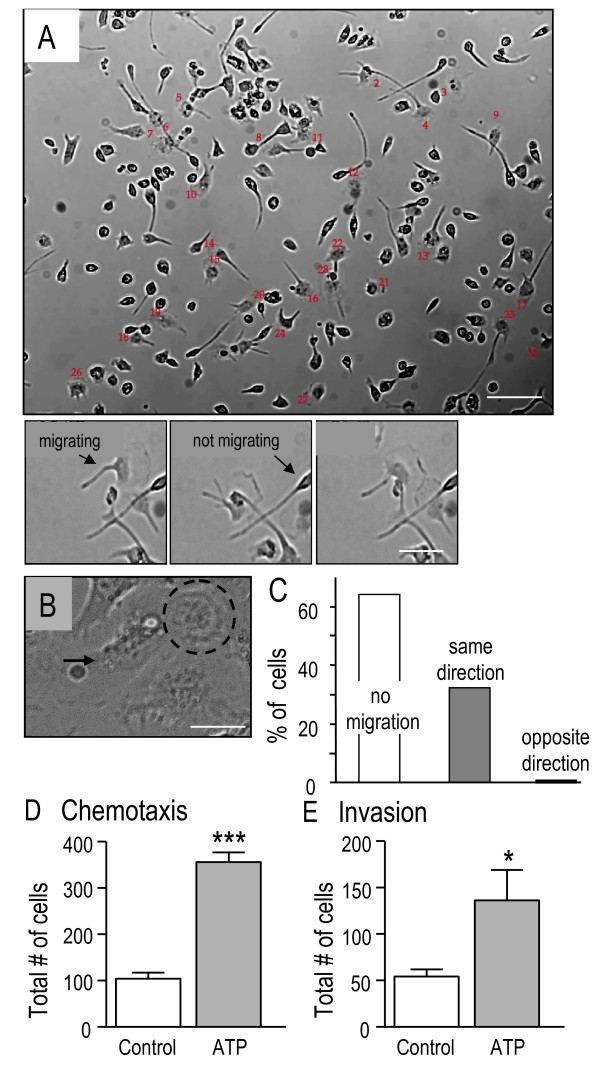
**Cultured rat microglia display chemokinesis, chemotaxis, and invasion.** (**A**) To monitor chemokinesis (random migration) in two dimensions, cultured rat microglia were imaged for 45 minutes under phase-contrast microscopy at 37°C and 5% CO_2_. A representative video frame capture, in which the numbered cells were tracked and analyzed (results in (c)). Inset: higher-magnification images show a lamella and extension of the leading edge in a migrating cell, but not in an immobile bipolar microglial cell. Scale bar: 80 μm (upper image), 40 μm (lower image). (**B**) Higher-magnification phase-contrast image showing a large ring-like structure in the lamella (circled), and a uropod at the trailing edge (arrow). Scale bar: 20 μm. (**C**) Each of the 28 microglial cells marked in (a) had a lamella at some time during the monitoring period. During a 10-minute examination period, these cells were classified as migrating in the direction of the lamella, in the opposite direction, or not moving. (**D**) Quantification of microglia transmigration through pores of 8 μm diameter in filters in the upper well of Transwell™ chambers. Microglia displayed chemotaxis toward 300 μM ATP added to the lower well. Values on bars are mean ± standard error of the mean (SEM) (*n* = 5). (**E**) Microglia invaded through Matrigel™-coated pores in the BioCoat Matrigel™ Invasion chambers, and invasion was increased by adding 300 μM ATP to the lower well. Values on bars are mean ± SEM (*n* = 6).

Immune cells can migrate by chemokinesis, chemotaxis or haptotaxis [13]; however, substrate degradation is an additional requirement for migration through basement membranes and the ECM. We found that untreated rat microglial cells under our standard culture conditions were highly migratory on glass coverslips. All microglia undergoing migration were polarized, with a fan-shaped lamella (Figure 1A,B,C) that was in constant motion, ruffling across the glass. Individual cells alternated between a migratory state, with lamellae and processes extended, and a nonmobile state with a condensed cell body, no lamella and often a bipolar appearance. Within 10 minutes after a lamella was observed, >35% of the microglia migrated and always in the direction of the lamella. Within a 30-minute period, some microglia changed direction but only after extending a new lamella at a different location and migrating in the direction of that lamella. Microglia that lacked a lamella (for example, bipolar cells) did not migrate during a 45-minute observation period. Within each lamella, a large, ring-like structure could often been seen under phase-contrast microscopy (Figure 1B). In addition to migrating along glass, microglia effectively trans-migrated (in three dimensions) through holes of 8 μm diameter in filters in the upper well of Transwell™ chambers, and displayed chemotaxis in response to ATP added to the lower well (Figure 1D). Importantly, microglia invaded to the underside of BioCoat Matrigel™ Invasion chambers, a process that requires degradation of Matrigel™, which coated the filter holes. Invasion was stimulated by an ATP gradient (Figure 1E).

### Microglia lamellae express podosomes and a podosome superstructure

**Figure 2:**
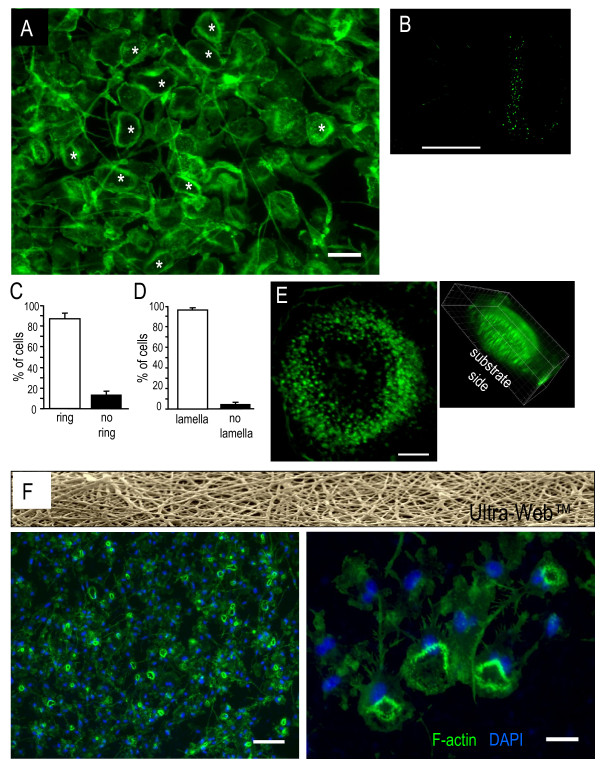
**Lamellae in microglia have a large F-actin ring, comprised of many small punctae.** F-actin was labeled with phalloidin (green) in all images. (**A**) Low-magnification image of many migrating microglia on a glass coverslip. Most lamellae have a large, donut-shaped F-actin ring (* indicates examples), which we call a podonut. Scale bar: 40 μm. (**B**) Higher-magnification, deconvolved image shows a podonut in the front half of a migrating microglial cell. Scale bar: 10 μm. (**C**), (**D**) F-actin rings were predominantly associated with microglial lamellae. Values expressed as mean ± standard error of the mean from three separate cultures. Most microglia with a lamella had a large F-actin ring (a podonut) (87 ± 5% of such cells (c)). As further demonstrated in Figure 3, the large ring was made up of many podosomes. When podosomes were present, they (and the large F-actin ring) were located in the lamella (95 ± 2% of such cells (d)). (**E**) The large F-actin rings contain hundreds of tiny punctae of F-actin. A representative deconvolved image of a single podonut shows that the large ring is composed of many tiny (<1 μm diameter) F-actin-rich punctae. Scale bar: 5 μm. Inset: a deconvolved three-dimensional (3D) reconstruction shows that the punctae of F-actin were located near the substrate-contacting cell membrane. (**F**) Microglia formed similar large, F-actin rings in their lamellae; nuclei stained with 4′,6-diamidino-2-phenylindole, (DAPI, blue) when cultured on Ultra-Web™, which is a 3D mesh of polyamide nanofibers (upper image). Right: higher magnification image of several microglia with large F-actin rings. Scale bar: 100 μm (left), 20 μm (right).

Phalloidin toxin staining of filamentous actin (F-actin) revealed a large donut-shaped ring in the lamella at the leading edge of most migrating microglia (Figure 2A). Higher-resolution deconvolved images (Figure 2B,E) show that this large F-actin ring is comprised of many submicron-sized punctae (which we found are podosomes, see below). Such F-actin-rich punctae were detected in 87 ± 5% of microglia that bore a lamella (Figure 2C), and when present the punctae were localized to the lamella (96 ± 2%; Figure 2D). Deconvolved images and 3D projections showed that the punctae were located near the substrate-contacting surface (Figure 2E). Expression of the large F-actin ring was not restricted to microglia growing on a glass substrate; it was prevalent in cells growing on a 3D mesh of polyamide nanofibers (Ultra-Web™; Figure 2F). However, because the imaging and deconvolution were not as clear on Ultra-Web™, the remaining immunocytochemistry was conducted on microglia growing on glass.

**Figure 3:**
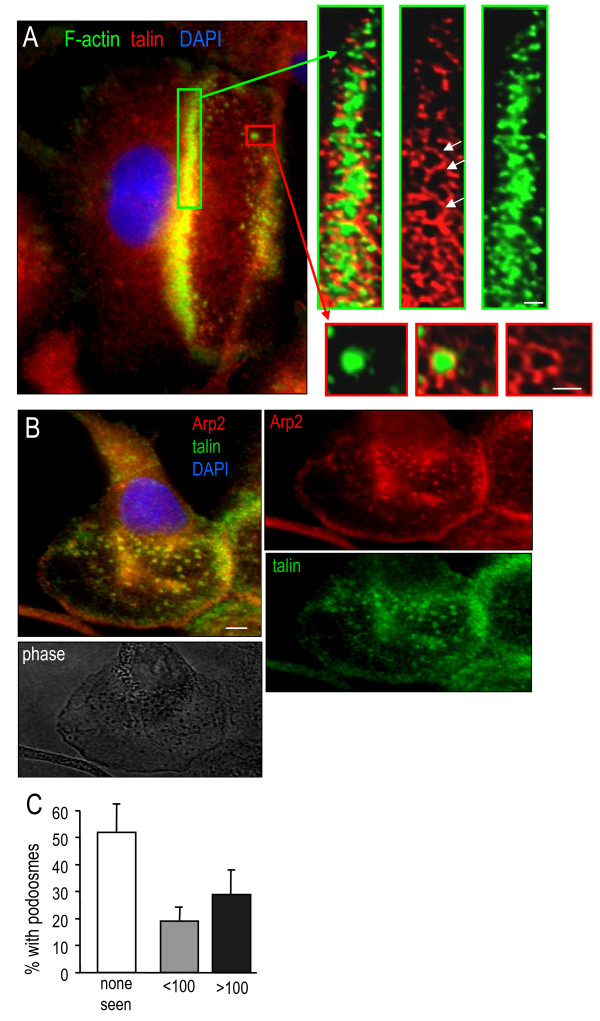
**The F-actin-rich punctae are podosomes.** (**A**) Microglia were stained for F-actin (phalloidin, green), the plaque protein talin (red), and the nuclear stain 4′,6-diamidino-2-phenylindole (DAPI; blue). A representative image of the large F-actin ring (podonut) in front of the nucleus shows co-localization with talin. Scale bar: 5 μm. Right: higher-magnification deconvolved images of the two boxed regions, color-separated and merged to show that the dense F-actin ring is comprised of many tiny punctae of F-actin and talin. Individual podosomes are seen as a core of F-actin, surrounded by a ring of talin (arrows show examples). Scale bar: 1 μm. (**B**) Microglia were immunostained for the plaque protein, talin (green), the actin nucleator actin-related protein 2 (Arp2; red), and labeled with the nuclear stain DAPI (blue). Arp2 is enriched in the podosome core, and the plaque protein talin identifies the ring. Lower image: under phase contrast, podosomes were often seen as dark punctae. (**C**) Prevalence of podosome expression in primary microglia (that is, percentage of cells with podosomes); >65 cells were analyzed per replicate from each of three cultures (mean ± standard error of the mean).

Small F-actin punctae are characteristic of both podosomes and the larger invadopodia, and distinguish them from other cell–matrix contacts, such as focal adhesion complexes [15]. In podosomes, the F-actin-rich core contains regulatory molecules (for example, the actin nucleator actin-related protein (Arp)2) and is surrounded by a ring containing structural proteins (for example, the plaque protein talin) [16,28] (see Discussion). In addition to F-actin, the large podosome superstructure in microglia (which we call a podonut) was enriched in talin (Figure 3A) and Arp2 (Figure 3B) staining. High-resolution deconvolution imaging showed that individual podosomes were tiny circular structures (0.5 to 1 μm diameter) with an F-actin core surrounded by a ring of talin, or a core of Arp2 surrounded by talin. We then quantified the prevalence of podosome expression by staining for F-actin and talin, and analyzing images at ≥40× magnification. Podosomes were detected in 48 ± 10% of microglia after at least 2 days of culturing, and 30 ± 9% of cells expressed >100 podosomes (Figure 3C). Microglia expressing >100 podosomes typically displayed the large F-actin-rich podonut in the lamella, while those with fewer podosomes had a more scattered distribution throughout the lamella or in other small membrane extensions.

### Microglial podosomes express several tyrosine phosphorylation-regulated components

**Figure 4:**
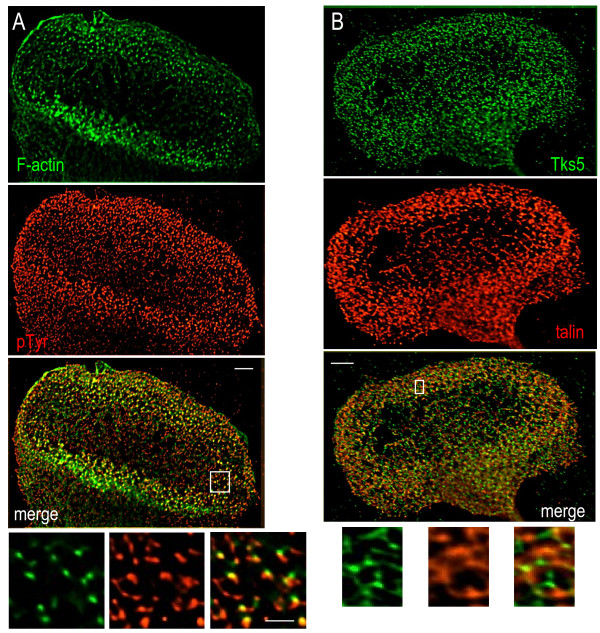
**Microglial podosomes are enriched in phosphotyrosine and the Src substrate Tks5.** Deconvolved, color-separated and merged images of entire podonuts are shown in the upper panels, and the boxed areas are shown magnified and color-separated below. Scale bar: 5 μm (upper), 2 μm (lower). (**A**) Immunostaining for phosphotyrosine residues (pTyr, red) is enriched in the F-actin-rich podonut (labeled with phalloidin, green). At higher magnification, some co-localization is seen. (**B**) Immunostaining shows tyrosine kinase substrate with five Src homology 3 domains (Tks5; green) in microglia podosomes together with the ring marker talin (red). The small punctae of Tks5 are often adjacent to the talin staining.

Podosome formation is stimulated by Src tyrosine kinase, and many components of podosomes and invadopodia are regulated by tyrosine phosphorylation (see Discussion); staining for phosphotyrosine (pTyr) is therefore often high in these structures. We found enriched pTyr staining in a punctate pattern at the leading edge of polarized microglia, and in the podonut (Figure 4A). At high resolution, pTyr labeling was round or elongated, some co-localized with the punctae of F-actin staining in the podosome core and some present adjacent to F-actin, suggesting localization to the ring as well. Outside the podonut, small punctae of pTyr staining were seen throughout the lamella.

One substrate of Src-mediated phosphorylation is tyrosine kinase substrate with five Src homology 3 domains (Tks5), which is enriched in invadopodia and podosomes in a few cell types [29,30] and is thought to act as an organizer in the initial stages of their assembly [30]. In microglia, Tks5 staining was enriched in podonuts and at high magnification appeared as small punctae, often in a ring-like arrangement and adjacent to talin (Figure 4B). Tks5 was also present in small punctae outside the podonut and throughout the lamella.

**Figure 5:**
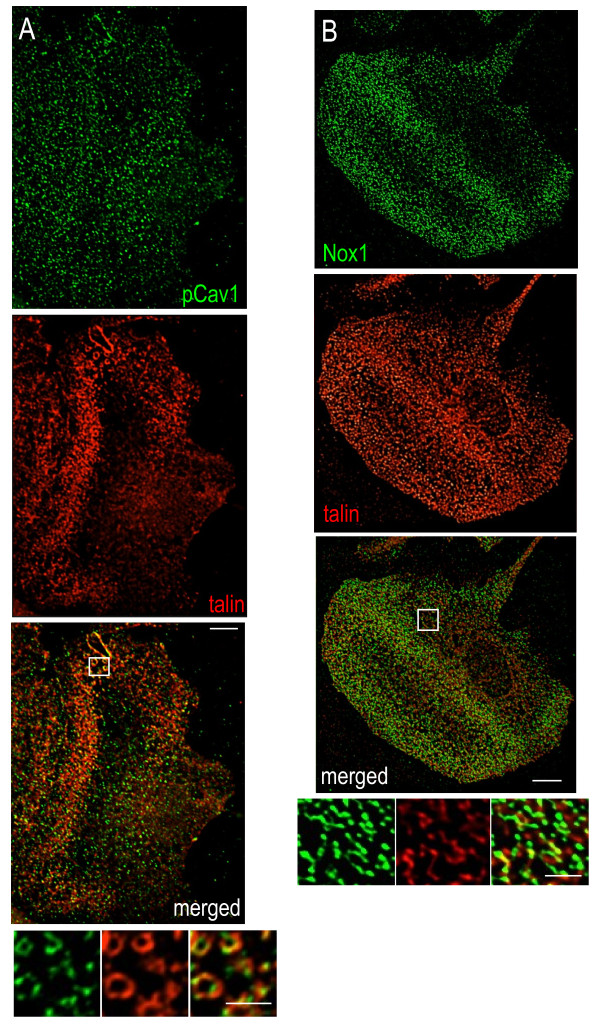
**Microglial podosomes are enriched in Tyr**^**14**^**-phosphorylated caveolin-1 and nicotinamide adenine dinucleotide phosphate oxidase 1(Nox1).** Deconvolved, color-separated and merged images of the lamellar region are shown for two microglia. The boxed areas are magnified in the images at the bottom. Scale bar: 5 μm (upper), 2 μm (lower). (**A**) Immunolabeling for tyrosine-phosphorylated caveolin-1 (p-Tyr^14^Cav1; green) and the podosome ring marker talin (red). (**B**) Immunolabeling for Nox1 (green) and talin (red). Note: Nox1 staining required methanol fixation.

Caveolin-1 (Cav1) is a major structural protein of caveolae [1], and can be phosphorylated at tyrosine 14 by Src-family kinases, including c-Src, Lyn and Hck [31,32]. p-Tyr^14^Cav1 was seen in podosomes in an ACTH-stimulated adrenal cell line [33]. In lamellae of microglia, p-Tyr^14^Cav1 was enriched in the podonut, and within individual podosomes it was in a ring-like pattern, closely associated with talin (Figure 5A).

Finally, we analyzed the distribution of Nox1 because of recent evidence that it is present in invadopodia and required for their formation in colon cancer cells [30] and that Nox1 is regulated by Src and Tks5 [34,35]. Nox1 was enriched in microglia podonuts, and at higher magnification appeared elongated and adjacent to, but rarely overlapping with, the talin staining (Figure 5B).

### Microglia with podosomes can degrade extracellular matrix molecules

**Figure 6:**
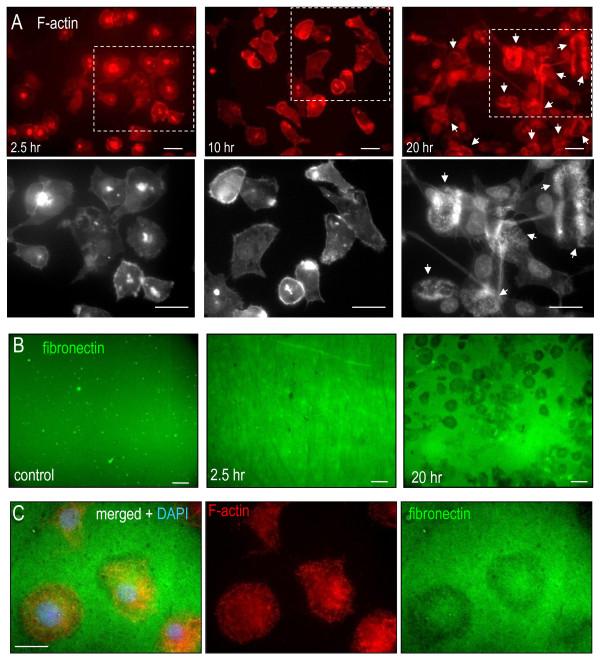
**Microglia with podosomes degrade the extracellular matrix molecule fibronectin.** (**A**) Development of F-actin superstructures (podonuts) with time after plating microglia on coverslips. Upper panels: microglia were labeled for the podosome core component F-actin (phalloidin, red). Podonuts in the lamellae were prevalent after 20 hours of culturing (examples shown by arrows), but not after 2.5 or 10 hours. Lower panels: higher-magnification grayscale images of the boxed regions. Scale bar: 40 μm (upper), 20 μm (lower). (**B**) Panels indicate the control condition at 20 hours without microglia (left), or 2.5 and 20 hours after adding microglia. Microglia were labeled for F-actin (phalloidin, red), and the substrate, fibronectin, was conjugated to AlexaFluor 488 (green). Representative images from three separate experiments. Scale bar: 40 μm. (**C**) Higher-magnification, color-separated and merged images showing microglia-sized areas of fibronectin degradation. Stains for F-actin and fibronectin were as in (b), and microglial nuclei were labeled with 4′,6-diamidino-2-phenylindole (DAPI, blue). Scale bar: 10 μm.

The key function usually ascribed to podosomes is ECM degradation, and the invasion assay (Figure 1E) shows that microglia can degrade Matrigel™. We next employed the standard test for podosome functionality, which is degradation of fluorescent-labeled substrate (fibronectin or gelatin) and consequent loss of fluorescence [21,36]. First, we determined that podonuts developed between 10 and 20 hours after cell plating, as seen from the F-actin staining (Figure 6A). This delay is not surprising, because microglia round up after being removed from the tissue culture flask and require some time before extending processes and becoming migratory (that is, 10 to 20 hours). To directly demonstrate substrate degradation, we then added microglia onto coverslips that were coated with a thin layer of fluorescent-labeled fibronectin, and incubated them for 2.5 to 20 hours. Fibronectin degradation produced many cell-sized regions of reduced fluorescence at 20 hours (Figure 6B) when podonuts were evident (Figure 6A). There was no degradation at 20 hours without microglia or at 2.5 hours with microglia. In the example shown at higher magnification (Figure 6C), three regions of fibronectin degradation (fluorescence loss) at 20 hours corresponded in size, shape and position to microglia.

**Figure 7:**
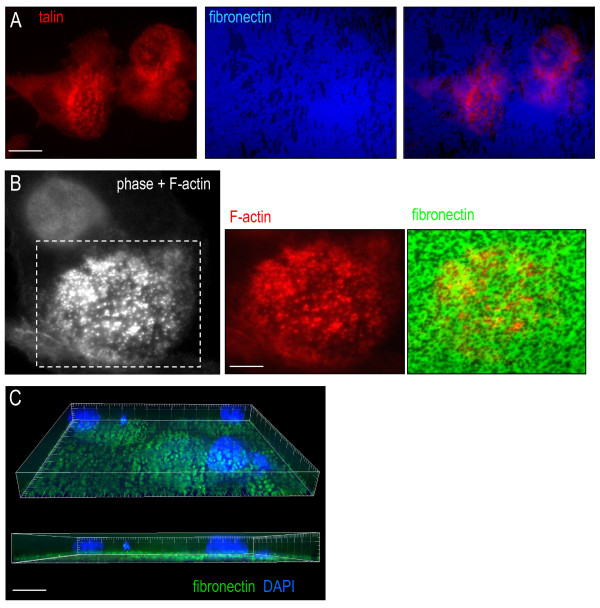
**Fibronectin degradation corresponds with podosomes.** (**A**) Microglia labeled with the podosome ring component, talin, showing two large clusters of podosomes (arrows). The cell at the right has a large ring of talin (a podonut). Fibronectin degradation (as in Figure 6) is indicated by loss of fluorescence (note: for better contrast, the AlexaFluor 488 was pseudo-colored as blue). Scale bar: 10 μm. (**B**) Co-localization of podosomes labeled for F-actin (phalloidin, red) with punctate regions of reduced fibronectin, labeled with AlexaFluor 488 (green). Left: combined phase-contrast and fluorescence image of the front of a microglia (nucleus is smooth circle), stained for F-actin (phalloidin), showing the podosome-rich lamella (boxed area). The colored images of the boxed area show F-actin, and the merged image with AlexaFluor 488-labeled fibronectin. Scale bar: 5 μm. (**C**) Fibronectin loss is not by phagocytosis. A high-resolution, deconvolved three-dimensional projection shows that punctae of fibronectin staining (green) are adjacent to the glass, rather than inside the microglia (4′,6-diamidino-2-phenylindole-stained nuclei, DAPI, blue). Scale bar: 10 μm.

Microglia plated on fibronectin often displayed a podonut or large clusters of podosomes that stained with F-actin and talin (Figure 7). Because our live imaging showed that microglia are highly mobile (Figure 1C,D,E) and podosomes have a high turnover rate (see Discussion), it was not surprising to observe trails of fibronectin degradation (Figure 7A). Some microglia were apparently less mobile, making it possible to correlate podosome clusters with similar-sized punctae of fibronectin degradation (Figure 7B). Importantly, we determined that loss of fluorescent fibronectin was not due to phagocytosis by microglia. That is, after adding Trypan Blue to quench the extracellular fluorescence, regions of remaining fluorescence were associated with cell bodies but the fluorescence signal was at the substrate–glass interface, not inside the microglia (Figure 7C). (Note: brighter fluorescence under the adherent cells was expected because of reduced access for the quenching agent, Trypan Blue.)

## Discussion

Microglial migration to sites of CNS damage is widely observed; however, the mechanisms regulating the ability of microglia to migrate through the neuropil and ECM are poorly understood. There are several salient findings in this study. Cultured rat microglia displayed spontaneous migration (chemokinesis) on bare or fibronectin-coated glass and a 3D mesh of polyamide nanofibers (Ultraweb™), and underwent ATP-stimulated chemotaxis through open or Matrigel™-coated filter holes. A second finding was that migrating microglia were polarized along the axis of movement, with a thin fan-shaped lamella at the leading edge. In nearly every migrating cell, the lamella contained a large F-actin-rich ring that was readily detected by staining with fluorescent phalloidin. Another finding was that each large ring was comprised of many small punctae (<1 μm diameter), which we showed to be classical podosomes having a core with F-actin and the F-actin nucleator, Arp2/3, surrounded by a ring of the structural protein talin. Podosomes in microglia were restricted to the lamella region, and were near the cell–substrate interface. The study also found that podosomes and their superstructures (podonuts) were readily detected in microglia growing on bare glass, a synthetic 3D mesh, or fibronectin. A fifth finding was that microglial podonuts and podosomes were also enriched in tyrosine-phosphorylated proteins and several molecules that are regulated by tyrosine kinases (Tks5, Cav1 and Nox1). Finally, podosomes formed in microglia within 20 hours after plating on fibronectin, at which time they degraded this ECM component; no degradation was seen at earlier times, however, when podonuts were lacking. At low magnification, fibronectin degradation often appeared as loss of fluorescence in cell-sized patches. At high magnification, fluorescence loss was seen as podosome-sized punctae (~1 μm) and trails of such punctae were seen, presumably occurring as microglia simultaneously migrated and degraded the fluorescent fibronectin.

Microglia undergo chemotaxis *in vitro*, responding to gradients of ATP, glutamate, and chemokines, such as CCL21 [37-39] – chemoattractants that are also thought to stimulate migration after damage *in vivo* (reviewed in [7]). Their ability to migrate can also be affected by ECM changes that occur after damage or disease. In the healthy adult CNS, the ECM (also called the peri-neuronal net) has high expression of glycosaminoglycans with unbranched polysaccharide chains that form space-occupying hydrated gels [40], as well as sulfated proteoglycans and tenascin glycoproteins [41]. The location of fibrous and elastic components is more restricted in the CNS; for example, fibronectin is mainly in the cerebrovasculature [42], and collagen is in basement membranes of the cerebrovasculature, dura mater and leptomeninges [41,43,44]. In addition, there are secreted matricellular proteins that are involved in cell–matrix interactions and signaling [12]. When the CNS is damaged, changes occur in the ECM and matricellular molecules. For instance, the matrix protein tenascin-R, which is anti-adhesive for activated microglia, is downregulated, which is thought to allow microglia to migrate into the injured site [45]. We recently reported that the anti-adhesive matricellular molecule SC1/hevin is upregulated in astrocytes and damaged axons in models of ischemic and hemorrhagic stroke [46]. Other expected changes after acute injury are disruption of the blood–brain barrier, edema, increased ECM hydration, and entry of plasma matrix proteins, including fibronectin and vitronectin [47]. Surprisingly, it is not known how microglia penetrate the ECM of either the healthy or damaged CNS.

In the 1980s, podosomes and invadopodia (often collectively called invadosomes) were identified and shown to possess the unique ability to both adhere to and degrade ECM components. These small, punctate structures at the ventral cell surface [17,18,28] differ from other cell adhesion structures in composition, architecture, dynamics and functions, and are now thought to contribute to cellular invasion in normal and pathological conditions (recently reviewed in [16,20,21,48]). Invadopodia in cancer cells appear as a small number of irregular punctae (~8 × 5 μm) near the nucleus and Golgi complex that can be stable for more than 1 hour [15,21]. Podosomes in cell lines [17,18,29,49] and the few normal cell types in which they have been found [50-52] are smaller (~0.4 × 1 μm), more numerous (often >100 per cell), more dynamic (lifetime of only a few minutes), and are sometimes in structured arrays [20,21]. Podosomes were recently seen *in vitro* in 3D cultures of macrophages [53] and transforming growth factor beta-stimulated endothelial cells [54], and in vascular smooth muscle cells of artery vessel walls *in vivo*[51]. The first evidence that podosomes degrade matrix was that osteoclasts from Src knockout mice lacked podosomes and the ability to resorb bone [55], and later it was found that loss of the podosome component Wiskott–Aldrich Syndrome protein prevented podosome formation [56] and matrix degradation [57,58]. Crucial signals for invadosome formation are cell attachment to a substrate [15,28], interactions between integrins and the ECM, and intracellular signaling initiated by activated Src [15,21], which is considered the key orchestrator. For instance, expressing constitutively active Src causes fibroblasts to express podosomes [16-18,48,59], while Src inhibition disrupts podosome formation in osteoclasts and macrophages [16,60,61]; conversely, inhibiting tyrosine phosphatases induces podosome formation in fibroblasts and monocytes [16,62]. Cells of myeloid lineage are apparently unique in their capacity to form podosomes spontaneously following cell adhesion [63,64], as was the case for microglia in the present study.

As sites of complex signal integration, invadosomes are enriched in many molecules. However, the current information derives from only a few cell types, and it is not clear whether the molecular profile reflects the tissue origin. For instance, gelsolin is apparently required for podosome formation in osteoclasts [48] but not in dendritic cells [65]. Podosomes are distinguished by their two-part architecture, with an F-actin-rich core surrounded by a prominent ring containing cell-type specific integrins [27,66] that anchor the podosome to ECM components (for example, fibronectin, vitronectin) [48] and adhesion-plaque proteins (talin, vinculin, paxillin) that link integrins to the actin cytoskeleton [67]. F-actin is important for podosome formation, and the core contains many actin regulatory molecules; that is, nucleators (Arp2/3 complex, formins), binding proteins (coronin, tropomyosin), filament crosslinkers (caldesmon, α-actinin) and polymerization activators (cortactin, Wiskott–Aldrich syndrome protein and its regulators) [16,48,63,64,66]. The most commonly used markers to identify podosomes are F-actin, talin, vinculin and Arp2/3 [16,28,48]. We found that podosomes in microglia had a classical structure; that is, they were tiny punctae (<1 μm diameter) present at the ventral cell surface, with a core containing F-actin and Arp2 and a ring containing talin. While podosomes are typically reported as isolated structures, we nearly always observed a superstructure (podonut) in the lamella in migrating microglia. This structure differs from osteoclasts, in which a belt of podosomes along the entire outer edge forms a sealing zone into which protons and proteases are secreted for bone resorption [68,69].

Tyrosine phosphorylation plays a central role in invadosomes, which are enriched in pTyr residues, protein tyrosine kinases (Src, Csk, Cbl, Pyk2) and adapter proteins (Tks5, Nck1, Nck2). After integrins are activated, Src can phosphorylate and activate other substrates [28] – including Tks5, which is one of the orchestrators of podosome formation [29,30,70]. Tks5 has previously been reported in invadopodia, but only in podosomes of phorbol ester-stimulated smooth muscle cells and myoblasts [71,72]. Tks5 can associate with cytoskeletal regulating proteins (neural Wiskott–Aldrich syndrome protein, dynamin, focal adhesion kinase) [73], dystroglycan [71], and matrix-degrading proteins (ADAMS-12, ADAMS-15 and ADAMS-19 [29], matrix metalloproteinase-9 [70]). In microglia, we found enrichment of pTyr residues and Tks5 in podonuts and individual podosomes; and while Tks5 was often adjacent to talin, it was also seen in other cellular locations. Another notable Src substrate is Cav1 [32,74], which can be phosphorylated at Tyr^14^ in response to growth factor signaling or oxidative stress [75,76], and can then regulate turnover of focal adhesions [77]. We found that the ring of podosomes in microglia was enriched in p-Tyr^14^Cav1. Cav1 has only been reported in invadopodia in three cell lines; that is, the nonphosphorylated form in two cancer cell lines [78,79], and the phosphorylated form in an ACTH-stimulated adrenal cell line [33]. Nox1 generates reactive oxygen species, which can activate protein kinases and inhibit protein tyrosine phosphatases [80]. Nox1 was found in invadopodia in human colon cancer cells [81,82], and its inhibition disrupted invadopodia formation [35,81]. Here, we show that Nox1 is enriched in microglial podosomes, which is especially notable because we have previously shown that reactive oxygen species production is a key mechanism whereby Kv1.3, SK3 and SK4 potassium channels control the capacity of activated microglia to kill neurons [22,23,83].

## Conclusion

Migrating microglia have a lamella that contains multi-molecular podosome structures and superstructures that can degrade components of the ECM. Microglial podosomes express several hallmark molecules seen in the few cell types in which they have been previously observed. The existence of podosomes in microglia has broad implications and might explain how these cells can migrate through the dense ECM in the brain; for example, in the developing brain, after damage, or in disease states when there is inflammation and matrix remodeling. In future, podosomes and their components might provide targets for diseases in which microglial accumulation is associated with negative outcomes; for example, stroke, hemorrhage, trauma, and spinal cord injury. The prospects are especially good because cancer cell invadopodia have several of the same molecules that are already being targeted for developing therapeutics.

## Abbreviations

ADAM, a disintegrin and a metalloprotease; Arp, actin-related protein; Cav1, caveolin-1; CNS, central nervous system; 3D, three-dimensional; ECM, extracellular matrix; FBS, fetal bovine serum; HPLC, high-performance liquid chromatography; MEM, minimal essential medium; Nox1, nicotinamide adenine dinucleotide phosphate oxidase 1; PBS, phosphate-buffered saline; pTyr, phosphotyrosine; Tks5, tyrosine kinase substrate with five Src homology 3 domains.

## Competing interests

The authors declare that they have no competing interests.

## Authors’ contributions

CV carried out the live imaging (Figure 1A,B,C), the immunocytochemistry and analysis in Figures 2, 3, 6 and 7, and defended an MSc thesis on this topic. TAS carried out the immunocytochemistry and analysis in Figures 4 and 5, helped conduct and interpret the migration assays (Figure 1d,e), defended an MSc thesis on the topic, and helped prepare the manuscript. LCS conceived and designed the project, obtained funding, supervised the work, and played a major role in interpreting results and preparing the manuscript. All authors read and approved the final manuscript.
